# Collection of Rare Peripheral Nerve Tumors—Insights from the German Registry

**DOI:** 10.3390/cancers16142599

**Published:** 2024-07-20

**Authors:** Nadja Grübel, Gregor Antoniadis, Anne-Kathrin Uerschels, Oliver Gembruch, Vera Marschal, Stefanie Deininger, Ralph König, Andrej Pala, Juliane Bremer, Nora F. Dengler, Melanie Reuter, Christian Rainer Wirtz, Maria Teresa Pedro

**Affiliations:** 1 Peripheral Nerve Unit, Department of Neurosurgery, BKH Günzburg at Ulm University, Lindenallee 2, 89312 Günzburg, Germany; gregor.antoniadis@uni-ulm.de (G.A.); dartmoorvera@aol.com (V.M.); s-deininger@web.de (S.D.); ralph.koenig@uni-ulm.de (R.K.); andrej.pala@uni-ulm.de (A.P.); rainer.wirtz@bkh-guenzburg.de (C.R.W.); maria-teresa.pedro@uni-ulm.de (M.T.P.); 2 Department of Neurosurgery, University Medicine Essen, Hufelandstraße 55, 45147 Essen, Germany; ann-kathrin.uerschels@uk-essen.de (A.-K.U.);; 3 Institute of Neuropathology, University Hospital RWTH Aachen, Pauwelstraße 30, 52074 Aachen, Germany; jbremer@ukaachen.de; 4 Department of Neurosurgery, Charité University of Berlin, Charitéplatz 1, 10117 Berlin, Germany; nora.dengler@helios-gesundheit.de; 5 Department of Neurosurgery, Helios Hospital Bad Saarow, Pieskower Str. 33, 15526 Bad Saarow, Germany; 6 Department of Neuroradiology, BKH Günzburg at Ulm University, Lindenallee 2, 89312 Günzburg, Germany; melanie.reuter@bkh-guenzburg.de

**Keywords:** rare peripheral nerve tumor, hybrid nerve sheath tumor, perineurioma, malignant nerve sheath tumor, neurofibromatosis

## Abstract

**Simple Summary:**

This study analyzes 61 rare tumors and lesions mimicking peripheral nerve tumors using data from the German Peripheral Nerve Tumor Registry. These cases exhibit significant heterogeneity with various morphological features and biological potentials, including malignant peripheral nerve tumors, perineuriomas, and hybrid nerve sheath tumors. This study underscores the challenges in preoperatively differentiating these rare entities from benign tumors using clinical and radiological methods alone. It highlights the importance of a multidisciplinary approach involving radiologists, neurologists, neuropathologists, and neurosurgeons for accurate diagnosis and effective treatment. The supplementary material offers valuable insights to help clinicians recognize these rare tumors early, improving patient outcomes.

**Abstract:**

The most common peripheral nerve tumors are of a benign nature and include schwannoma or neurofibroma. In rare cases, other tumors or non-tumorous lesions can mimic peripheral nerve tumors clinically or radiologically. Based on data from the multicentric German Peripheral Nerve Tumor Registry (PNTR), which encompasses current information on 315 surgically treated patients from three high-volume centers, we present 61 cases of rare tumors and lesions that mimic tumors associated with peripheral nerves. This cohort displays considerable heterogeneity, featuring a broad spectrum of morphological features and biological potentials. Histopathological diagnoses include various intrinsic peripheral nerve tumors such as malignant peripheral nerve tumors (MPNSTs) (*n* = 13), perineurioma (*n* = 17), and hybrid nerve sheath tumors (HPNSTs, comprising schwannoma/perineurioma and schwannoma/neurofibroma) (*n* = 14), as well as atypical neurofibromatous neoplasm with unknown biological potential (ANNUBP) (*n* = 1). Additionally, the cohort encompasses extrinsic tumorous lesions like lymphoma (*n* = 3), lymphangioma (*n* = 2), hemangioma (*n* = 2), solitary fibrous tumor (*n* = 2), metastatic disease (*n* = 1), and single cases of other rare tumor entities (*n* = 6). An overview of the underlying pathology, imaging features, and clinical presentation is provided, with a brief description of each entity. A definitive preoperative differentiation between benign peripheral nerve tumors and rare intrinsic and extrinsic tumors is often not possible. Clinical examination and subtle imaging clues can at least indicate the possibility of a rare entity. The basic requirement is close cooperation between radiologists, neurologists, neuropathologists, and neurosurgeons at a specialized center to develop a multidisciplinary concept and offer the patient the best therapeutic approaches.

## 1. Introduction

Rare peripheral nerve tumors represent both diagnostic and therapeutic challenges [1,2,3,4,5,6,7,8]. To date, the literature consists only of a few case reports and small series without reference to the frequency of these rare lesions. Distinguishing these lesions can be challenging, as they may present clinically and radiologically, like schwannoma and neurofibroma [1,9]. The rare peripheral nerve lesions in focus encompass intrinsic peripheral nerve tumors (PNTs): hybrid nerve sheath tumors (HPNSTs), malignant peripheral nerve sheath tumors (MPNSTs), perineurioma, and atypical neurofibromatous neoplasm with uncertain biological potential (ANNUBP) and extrinsic tumorous (metastatic tumor, lymphoma, amyloidoma, hemangioma, angioma, solitary fibrous tumors, lymphangioma, and desmoid tumors) and non-tumorous lesions. The multicentric peripheral nerve tumor registry [10] provides multicentric data on rare tumorous lesions for the first time. This article aims to provide an overview of the clinical, imaging, and pathological features of uncommon lesions associated with peripheral nerves, emphasizing characteristics that set them apart from common peripheral nerve tumors like schwannoma and neurofibroma.

Our study group analyzes the findings and collects the experience of 61 rare peripheral nerve pathologies identified among our surgical series of 315 patients. Representative imaging is provided, and a brief review of each lesion and its recommended therapy is discussed.

## 2. Materials and Methods

### 2.1. The Peripheral Nerve Tumor Register (PNTR)

In 2015, the Multicentric Peripheral Nerve Tumor Registry (PNTR) was established in Germany, allowing for systematic analysis of patients with benign, malignant, and other rare tumor entities associated with peripheral nerves. As of now, there are only few data on rare peripheral nerve tumors existing in Europe. Patient characteristics (such as age and sex), disease details (affected nerve, tumor location), surgical treatment (type of treatment, pre- and postoperative symptoms), and the diagnosis of neurofibromatosis (NF) were retrospectively analyzed. Histopathological examination was performed in every case. To better structure our findings, the histological results were divided into two groups: (1) intrinsic tumors, summarizing all lesions originating from the peripheral nerve itself, and (2) extrinsic lesions, collecting all lesions of extraneural origin.

### 2.2. Imaging: Magnetic Resonance Imaging and Ultrasound

**Figure 1:**
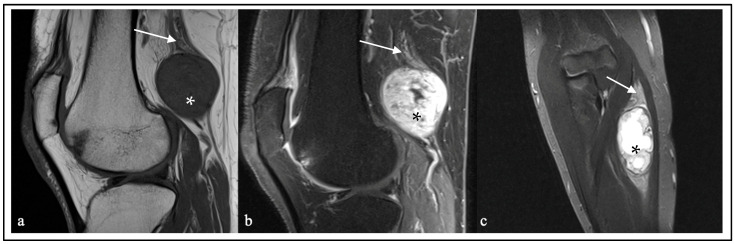
MRI features of hybrid nerve sheath tumors. (**a**) T1 MRI shows a homogeneous hypointense (*) mass associated with the sciatic nerve (arrow). There is no evidence of infiltration into adjacent tissues or a perilesional edema. (**b**) A heterogeneous contrast enhancement of the mass (*). (**c**) T2 STIR sequence shows an inhomogeneous, partially cystic mass (*) originating from the cutaneous antebrachial medial nerve (arrow).

**Figure 2:**
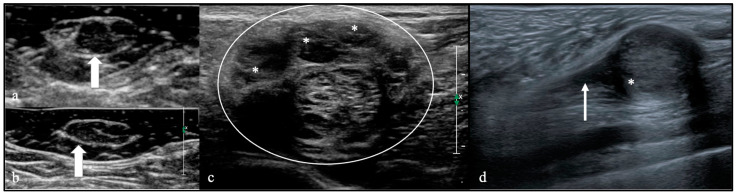
Ultrasound presentation of rare peripheral nerve tumors (PNTs). (**a**,**b**) Hypoechoic, enlarged fascicles (white arrow) in a patient with perineurioma, representing Type A in sonotypification. (**c**) Ultrasound image of a patient with lymphoma manifestation on the sciatic nerve shows a mass with dense, giant fascicles (*) representing Type B. (**d**) Ultrasound image of a Type C hybrid nerve sheath tumor (schwannoma/perineurioma) originating from the cutaneous antebrachial medial nerve (arrow), with no fascicle structures found in the mass (*).

Imaging evaluation was performed using preoperative magnetic resonance imaging (MRI) and ultrasound (Figure 1 and Figure 2). Based on the publication by Pedro et al. [9], ultrasonographic characteristics were divided into three groups: (1) hypoechogenic with enlarged fascicles (Group A); (2) exhibiting dense, giant fascicles (Group B); and (3) lacking distinguishable fascicle structure, with an irregular mass showing cystic, hypoechogenic, or isoechoic to hyperechogenic parts (Group C). These groups were used to classify the ultrasonographic patterns in the herein-described rare entities. Ultrasound examinations were not conducted for all patients, resulting in missing data. The follow-up examinations were performed according to clinical standards.

### 2.3. Data Collection

The PNTR currently holds up-to-date data of 315 surgically treated patients who received treatment from January 2015 to January 2024 at one of the three high-volume recruiting centers: the Department of Neurosurgery for the University of Ulm, peripheral nerve unit in Günzburg; the Department of Neurosurgery at Charité University Hospital, Berlin; and the Department of Neurosurgery, University Hospital Essen. In case of histopathological doubt, reference histopathological analysis was performed either by the Department for Neuropathology at the University Hospital in Aachen or by an institute for Pathology specialized in this entity in Germany.

Patients with histological diagnoses of schwannoma, neurofibroma, and intraneural lipoma were excluded from this present analysis. Twelve patients were dropouts due to missing tumor detection after histopathological diagnosis. All patients provided written permission. This study received approval from our local ethics committees in Ulm (Nr. 249/17) and Berlin (EA4/058/17) and is registered in the German Trials Registry (www.drks.de, accessed on 29 April 2021).

## 3. Results

In 61 cases, we considered the lesion as a rare PNT. In relation to the entire collective, consisting of 315 patients, this accounts for 19%. After subclassifying the cohort of rare PNTs, the percentage of extrinsic lesions was 5%, while 14% were intrinsic tumors in our series. In total, 41% of affected patients were male (*n* = 25), and 59% (*n* = 36) were female, with a mean age of 42.5 years (range 11–78 years). The upper extremity was affected in 31 cases (51%), while the lower extremity was affected in 30 cases (49%). The left side was slightly more affected than the right (51% vs. 49%). The most affected neural structures were the sciatic nerve (23%), the brachial plexus (15%), and the median nerve (13%).

### 3.1. Histological Examination

For this analysis of rare tumor entities associated with peripheral nerves, we established a classification of intrinsic and extrinsic lesions. The most frequently represented intrinsic tumors were perineurioma (*n* = 17) and HPNSTs (*n* = 14), including 12 patients of the subgroup schwannoma/neurofibroma (Figure S1) and 2 patients of the subgroup schwannoma/perineurioma (Figure S2). Lymphoma (*n* = 3) and hemangioma (*n* = 2) were representatives for the extrinsic lesions. Histopathological diagnoses of all 61 patients are summarized in Table 1. In case of uncertain pathological diagnosis, reference histopathological analysis was performed either by the Department for Neuropathology at the University Hospital in Aachen or by an institute for Pathology specialized in this entity in Germany.

### 3.2. Neurological Symptoms and Pain

Clinical symptoms were usually caused by local nerve compression and/or infiltration, with the most common symptom being pain. Preoperative pain symptoms were divided into pain at rest (*n* = 24, 39%) and stress-induced pain (*n* = 41, 67%). Postoperatively, only 19 patients (31%) described residual pain. Preoperative motoric deficits occurred in 46% (*n* = 28) and sensory deficits in 51% (*n* = 31). Preoperative motor and sensory deficits did not resolve after surgical treatment in all cases (100%). In contrast to the other intrinsic lesions, patients with HPNSTs and ANNUBP (Figure S5) did not have any motoric deficits pre- and postoperatively. Table 1 summarizes the clinical presentation of rare PNTs.

### 3.3. Imaging Findings

The MRI images were examined regarding the size of the lesion, contrast enhancement, and infiltration of neighboring tissue. Additionally, the T2 behavior of the lesions was categorized into heterogeneous and homogeneous, supplemented by whether cystic components were present (Figure 1). The maximum lesion diameter varied from 7 to 190 mm, with perineurioma and lymphoma being, on average, the largest lesions. Except for one patient with capillary hemangioma (Figure S14) and one patient with angiomatosis (Figure S13), all tumors and lesions were contrast-enhanced on MRI. In this cohort, the invasion of adjacent tissue was frequently observed, especially in patients with MPNSTs (*n* = 3, 23%) (Figure S3) and extrinsic lesions (for example, metastatic tumor (*n* = 1) (Figure S6), lymphoma (*n* = 2) (Figure S7), and desmoid tumor (*n* = 1) (Figure S8)). These lesions are often heterogeneous on MRI T2 sequences, which is an underestimated feature, especially the intrinsic lesions. Occasionally, they may exhibit cystic structures or hemorrhages, which can provide clues to malignancy. Perineurioma are characterized in MRI and ultrasound by enlarged, contrast-enhanced fascicles and frequent caliber changes along the course of the nerve (Figure S4). The longitudinal extension is often quite extensive. Lymphangiomas present with a honeycomb-like structure, making them visually diagnosable (Figure S16). After evaluating the ultrasound examinations, most lesions exhibited a typical hypoechogenic behavior (Figure 2). Table 2 outlines the MRI characteristics of uncommon peripheral nerve lesions, while Table 3 delineates the ultrasound traits (Figure S11).

### 3.4. Surgical Treatment and Outcome

Microsurgical gross total resection was performed in the cases of HPNSTs, ANNUBP, hemangioma (Figure S12), SFTs (Figure S10), EHE (Figure S15), and Myopericytoma (Figure S9). Due to invasive growth or to spare neurological function, surgery was limited to only a biopsy (*n* = 23, 38%) or partial resection (*n* = 12, 19%) for diagnosis confirmation. The mean follow-up was 26 months. Adjuvant treatment was individually indicated, according to local tumor board decisions. No recurrence was observed in the HPNST group. Table 4 demonstrates the extent of removal, adjuvant treatment, and follow-up in patients with rare peripheral nerve lesions.

### 3.5. Dropouts

Among the entire cohort of 315 patients, 12 cases (3.8%) presented lesions that were clinically and, based on imaging, highly suspicious of NSTs. These cases give an overview of lesions mimicking PNTs. In six cases, only a biopsy was performed; in three cases, complete resection was indicated; in two cases, only neurolysis was performed due to intraoperative absence of tumor tissue. Histopathological diagnosis revealed non-tumorous lesions, including plexus neuritis (*n* = 1), reactive perineural proliferation (*n* = 1), no pathological finding (neither neoplastic nor inflammable) (*n* = 3), ectatic subclavian vein (*n* = 1), thrombosed aneurysm of the ulnar artery (*n* = 1), neuroma (*n* = 1), chronic neuropathy (*n* = 2), ganglion cyst (*n* = 1), and nonabdominal endometriosis cyst (*n* = 1). Consequently, these patients were considered dropouts. In all cases, the preoperative diagnostic work-up led to misinterpretation.

### 3.6. Supplementary Material

In the Supplementary Material, an overview of the imaging features and a brief case presentation are provided. Due to the rarity of these lesions, the included imaging and intraoperative images hold significant value, as some of the lesions can be diagnosed visually.

## 4. Discussion

In our surgical series, 19% of lesions were considered “rare” and associated with peripheral nerves. If we exclude the intrinsic rare PNTs, our cohort’s percentage of non-neural lesions is 5%. From this, it can be inferred that these rare tumors occur more frequently than previously thought. However, there are currently no data available on their incidences.

### 4.1. Demographic Data

The rare entity cohort included 25 (41%) male and 36 (59%) female patients, with an average age of 43 (ranging from 11 to 78 years). Conversely, the cohort of 242 patients diagnosed with schwannoma and neurofibroma included 133 (55%) male and 109 (45%) female patients, with an average age of 48 (ranging from 10 to 85 years). Our findings indicate that patients with rare lesions are younger and more frequently female. The sciatic nerve was the most affected nerve in the rare PNT cohort, whereas, in the cohort of schwannoma and neurofibroma, the brachial plexus was the most affected nerval structure. Other authors found that lymphoma and perineurioma manifestation seems to show a predilection for the sciatic nerve [5,7,11,12,13,14]. It remains unclear why the sciatic nerve is most commonly affected. Neurofibromatosis type 1 (NF1, *n* = 4), type 2 (NF2, *n* = 1), schwannomatosis (*n* = 2) and legius syndrome (*n* = 1) were detected in 13% of the rare disease cohort. In contrast, they were found in only 8% of the benign nerve tumor cohort, as expected.

### 4.2. Clinical Symptoms

In our series of rare PNTs, the clinical symptoms consisting of motoric and sensory deficits did not resolve after surgery, quite contrary to the cohort of benign peripheral nerve tumors. Pain was the only symptom of relevance after surgery. Patients benefited from surgery in the domain of pain, which is essential for quality of life. Contrary to previous beliefs, patients with perineurioma experience pain disturbances, including rest pain (*n* = 3, 18%) and stress pain (*n* = 8, 47%) [14]. These symptoms improved after surgery involving biopsy and extensive epineuriotomy, suggesting a plausible explanation for the improvement [5]. Patients with MPNSTs have the highest rate of resting pain preoperatively at 69%, which persists postoperatively.

### 4.3. Surgery

The objective of surgical intervention for a solitary peripheral nerve tumor should encompass complete resection while safeguarding both motor and sensory function and alleviating tumor-induced pain. This is predominantly achieved through microsurgical resection employing nerve stimulation. Biopsy, even when guided by ultrasound, poses a substantial risk of temporary or permanent nerve damage [15]. Should complete resection with preservation of function be difficult to achieve, an open biopsy or a subtotal resection sparing functional fascicles may be considered for diagnostic purposes. In cases of perineurioma, complete resection is discouraged to maintain residual nerve function. For diagnostic confirmation, an open biopsy of a motoric silent fascicle is recommended, followed by a long-distance epineuriotomy to decompress hypertrophic fascicles, constituting the preferred surgical approach [5]. Broad surgical indications and prompt resection of suspicious masses in NF1 patients can aid in the early detection of malignant transformation [16,17].

### 4.4. Pathological Analysis

The appropriate diagnosis relies on pathological examination. While peripheral nerve sheath tumors were historically diagnosed solely based on their histopathological, i.e., morphological and immunohistochemical, features, the significance of molecular diagnostic approaches becomes increasingly evident. Furthermore, even the histopathological distinction of tumor entities has broadened over the last years. For example, with the revision of the fourth edition of the WHO classification of tumors of the central nervous system (CNS), hybrid nerve sheath tumors (HNSTs) were added in 2016 [18]. More recently, in 2021, the fifth edition of the WHO classification of tumors of the CNS has introduced atypical neurofibromatous neoplasms of uncertain biological potential (ANNUBP) in the setting of NF1 [19]. Regarding the advances in molecular diagnostics, methylation profiling, for example, currently allows for differentiation between the major nerve sheath tumor entities [20], although some uncertainties remain, especially for the novel entity’s hybrid NSTs and ANNUBP and likewise for atypical neurofibromas and certain subtypes like epithelioid MPNST. According to the literature, the combination of schwannoma/perineurioma is the most frequent in BPNSTs [19]. This differs from the observation in our cohort; i.e., in our series of 14 patients with HPNSTs, the majority showed the combination of schwannoma and neurofibroma (*n* = 12). Interestingly, patients in this cohort diagnosed with HPNSTs did not experience pain relief after surgical removal [3]. In our cohort, HPNSTs tended to be larger than typical schwannomas and neurofibromas. The mean maximum diameter of all HPNSTs was 40.8 (SD 39.6) mm (Table 1), compared to 30 (SD 21.6) mm in the cohort of schwannomas and neurofibromas. We hypothesize that this larger tumor size leads to increased preoperative pain due to compression and damage to surrounding structures. The range of potential non-neural lesions affecting peripheral nerves is broad but exceedingly rare. Often, these lesions are indistinguishable from peripheral nerve tumors in preoperative imaging and sometimes even histologically, and therefore, they particularly benefit from careful postoperative pathological assessment, including recent advances in molecular diagnostics. One such example is synovial sarcoma, which can resemble nerve sheath tumors clinically and histologically [21,22] and requires molecular diagnostics for verification.

### 4.5. Imaging

Currently, there is no standardized preoperative imaging protocol for peripheral nerve tumors (PNTs). Gadolinium-enhanced MRI T1, T2, and ADC sequences are valuable for preoperatively assessing malignancy and lesion origin, aiding in more precise surgical planning. Monitoring entities like perineurioma or desmoid tumors via imaging is challenging due to their growth patterns. Although volumetry would be beneficial, standardization is lacking. The absence of standardized sequences, such as T2, often hinders comparison with preoperative MRI images, which provides crucial tumor information. High-resolution nerve sonography serves various purposes: gaining an overview of existing pathology, preoperative planning of incision paths, and monitoring large superficial lesions like perineurioma throughout the disease course. MRI and nerve sonography are not competing but complementary modalities, each enhancing the other’s diagnostic capabilities [8,15,23]. The high number of dropouts highlights the difficulty of preoperative diagnosis once again, underscoring the need for standardized imaging.

### 4.6. Possible Indicators for Malignisation

Indicators of malignant processes could include rapid growth, including a palpable tumor mass, swift clinical deterioration with new-onset paresis and sensory lesions, and unbearable pain. In MRI, indicators may encompass large tumors (>5 cm) with cystic components, perilesional edema, and infiltrating of neighboring structures. Additionally, high FDG uptake in FDG PET/CT can serve as a valuable tool for detecting malignancy early [24,25,26]. A well-known risk factor for malignisation is the presence of NF 1. Patients develop multiple neurofibromas over the course of the disease with a lifetime risk of malignant transformation into malignant peripheral nerve sheath tumors (MPNSTs) in 8–15% [16,27,28].

### 4.7. Limitations

This cohort consists of a highly diverse array of tumors and lesions, with many entities being represented by only one case. Due to the limited number of patients, statistical analysis is not possible, and thus, the analysis remains purely descriptive. Attempts were made to standardize the comparison of these rare tumors and lesions as comprehensively as possible and to provide representative imaging and clinical data.

## 5. Conclusions

In our experience, the prevalence of rare peripheral nerve tumors (PNTs) exceeds expectations, particularly following new histological diagnoses such as hybrid nerve sheath tumors. Recognizing their existence is vital for tailoring surgical approaches and treatment strategies and for considering adjuvant therapy when warranted. Our experience shows that conclusively distinguishing between benign PNTs and rare intrinsic and extrinsic tumors during preoperative diagnostics may be challenging. However, thorough clinical examinations and subtle imaging cues can at least hint at the presence of a rare entity.

## Figures and Tables

**Table 1:** Summary of the neurological symptoms and pain of patients with rare peripheral nerve lesions. Next to the number of patients with each symptom appears the percentage of symptoms in each histopathological diagnosis: T1 preoperative status, T2 postoperative status.

Pathological Diagnosis	Neurological Symptoms				Pain	
	Motor Deficits ^T1^	Sensor Deficits ^T1^	Motor Deficits ^T2^	Sensor Deficits ^T2^	Rest Pain ^T1^	Stress Pain ^T1^
Intrinsic PNT						
HPNST (*n* = 14)		4 (29%)		4 (29%)	5 (36%)	11 (79%)
MPNST (*n* = 13)	6 (46%)	7 (54%)	7 (54%)	10 (77%)	9 (69%)	9 (69%)
Perineurioma (*n* = 17)	16 (94%)	10 (59%)	16 (94%)	10 (59%)	3 (18%)	8 (47%)
ANNUBP (*n* = 1)					1 (100%)	1 (100%)
Extrinsic PNT						
Metastasis (*n* = 1)		1 (100%)		1 (100%)	1 (100%)	1 (100%)
Lymphoma (*n* = 3)	2 (67%)	2 (67%)	2 (67%)	2 (67%)	1 (33%)	2 (67%)
Amyloidoma (*n* = 1)	1 (100%)	1 (100%)	1 (100%)	1 (100%)	1 (100%)	1 (100%)
Hemangioma (*n* = 2)	1 (50%)	2 (100%)	1 (50%)	2 (100%)	1 (50%)	1 (50%)
Angiomatosis (*n* = 1)		1 (100%)		1 (100%)		1 (100%)
Capillary hemangioma (*n* = 1)	1 (100%)	1 (100%)	1 (100%)	1 (100%)		
EHE (*n* = 1)						1 (100%)
SFT (*n* = 2)					2 (100%)	2 (100%)
Lymphangioma (*n* = 2)		2 (100%)		1 (50%)		1 (50%)
Desmoid (*n* = 1)	1 (100%)	1 (100%)	1 (100%)	1 (100%)		1 (100%)
Myopericytoma (*n* = 1)						1 (100%)

**Table 2:** Summary of MRI features of rare peripheral nerve lesions. Size corresponds to the maximum lesion diameter. The lesion component was evaluated in T2 MRI. Next to the number of cases appears the percentage of imaging findings in each lesion. * Largest lesion diameter; ih, inhomogeneous; h, homogeneous.

Diagnosis	MRI Features			Lesion Component in T2		
	Size * (mm), Range, Mean	Contrast Enhancement (*n*, %)	Invasion (*n*, %)	Cystic (*n*, %)	Ih (*n*, %)	H (*n*, %)
Intrinsic PNTs						
HPNST (*n* = 13)	19–76 (41)	13 (100%)	0	5 (38%)	12 (92.3%)	1 (8%)
HPNST (S/NF) (*n* = 11)	19–76 (40)	11 (100%)	0	4 (36%)	10 (91%)	1 (9%)
HPNST (S/P) (*n* = 2)	23–71 (47)	2 (100%)	0	1 (50%)	2 (100%)	0
MPNST (*n* = 13)	27–96 (57)	13 (100%)	3 (23%)	7 (53%)	12 (92%)	1 (8%)
Perineurioma (*n* = 17)	15–190 (72)	17 (100%)	0	14 (82%) Enlarged fascicles and/or change in calibers		
ANNUBP (*n* = 1)	52	1 (100%)	0	0	1 (100%)	0
Extrinsic PNTs						
Metastatic tumor (*n* = 1)	80	1 (100%)	1 (100%)	1 (100%)	1 (100%)	0
Lymphoma (*n* = 3)	21–120 (81)	3 (100%)	2 (67%)	0	3 (100%)	0
Amyloidoma (*n* = 1)	29	1 (100%)	0	0	0	1 (100%)
Hemangioma (*n* = 1)	140	1 (100%)	1 (100%)	1 (190%)	1 (100%)	
Angiomatosis (*n* = 1)	12	0	0	0	0	1 (100%)
Capillary hemangioma (*n* = 1)	7	0	0	0	0	1 (100%)
EHE (*n* = 1)	28	1 (100%)	1 (100%)	1 (100%)	1 (100%)	
SFT (*n* = 2)	29–57 (43)	2 (100%)	0		2 (100%)	
Lymphangioma (*n* = 2)	48–82 (65)	2 (100%), honeycomb-like	0		2 (100%), honeycomb-like	0
Desmoid (*n* = 1)	49	1 (100%)	1 (100%)	0	0	1 (100%)
Myopericytoma (*n* = 1)	10	1 (100%)	0	0	0	1 (100%)

**Table 3:** Summary of ultrasound features of rare PNTs. Sonotypification was performed after Pedro et al.’s classification [9].

Histopathological Diagnosis	Ultrasound					
	Echogenicity			Type		
	Iso (*n*, %)	Hypo (*n*, %)	Hyper (*n*, %)	A (*n*, %)	B (*n*, %)	C (*n*, %)
Intrinsic PNTs						
HPNST (S/NF) (*n* = 3)	1 (33%)	2 (67%)				3 (100%)
MPNST (*n* = 7)	1 (8%)	6 (46%)				7 (100%)
Perineurioma (*n* = 10)	2 (20%)	8 (80%)		10 (100%)		
ANNUBP (*n* = 1)		1 (100%)				1 (100%)
Extrinsic PNTs						
Lymphoma (*n* = 1)	1 (100%)				1 (100%)	
Amyloidoma (*n* = 1)		1 (100%)		1 (100%)		
Hemangioma (*n* = 1)		1 (100%)				1 (100%)
Angiomatosis (*n* = 1)		1 (100%)		1 (100%)		
Capillary hemangioma (*n* = 1)		1 (100%)		1 (100%)		
EHE (*n* = 1)		1 (100%)				1 (100%)

**Table 4:** Summary of adjuvant treatment, outcome, and follow-up of patients with rare peripheral nerve lesions. SD: stable disease, R: remission, P: progression, LFU: lost follow-up, NR: no recurrence, D: death.

Histopathological Diagnosis	Extent of Removal			Adjuvant Treatment	Outcome	Follow-Up
	Biopsy (*n*, %)	Partial Resection (*n*, %)	Total Resection (*n*, %)			(Mean, Months)
Intrinsic PNTs						
HPNST (*n* = 14)			14 (100%)	none	NR, *n* = 14	12
MPNST (*n* = 13)	2 (15%)	7 (54%)	4 (31%)	individual	R, *n* = 1	29
					SD, *n* = 3	
					P, *n* = 1	
					D, *n* = 4	
					LFU, *n* = 4	
Perineurioma (*n* = 17)	17 (100%)			none	SD, *n* = 17	39
ANNUBP (*n* = 1)			1 (100%)	no	NR, *n* = 1	6
Extrinsic PNTs						
Metastatic tumor (*n* = 1)		1 (100%)		individual	D, *n* = 1	6
Lymphoma (*n* = 3)	1 (33%)	1 (33%)	1 (33%)	individual	SD, *n* = 2	48
					LFU, *n* = 1	
Amyloidoma (*n* = 1)	1 (100%)			Antibody therapy	SD, *n* = 1	3
Hemangioma (*n* = 2)			2 (100%)	no	NR, *n* = 2	31
Angiomatosis (*n* = 1)		1 (100%)		no	SD, *n* = 1	18
Capillary hemangioma (*n* = 1)	1 (100%)			no	SD, *n* = 1	3
EHE (*n* = 1)			1 (100%)	no	SD, *n* = 1	3
SFT (*n* = 2)			2 (100%)	no	NR, *n* = 2	9
Lymphangioma (*n* = 2)	1 (50%)	1 (50%)		no	SD, *n* = 2	12
Desmoid (*n* = 1)		1 (100%)		Sorafenib	SD, *n* = 1	18
Myoperizytoma (*n* = 1)			1 (100%)	no	NR, *n* = 1	3

## Data Availability

The authors will make the raw data supporting this article’s conclusion available upon request.

## Supplementary Materials

The following supporting information can be downloaded at https://www.mdpi.com/article/10.3390/cancers16142599/s1, Figure S1: Hybrid nerve sheath tumor (Schwannoma/Neurofibroma). Figure S2: Hybrid nerve sheath tumor (Schwannoma/Perineurioma). Figure S3: Malignant peripheral nerve sheath tumor. Figure S4: Perineurioma. Figure S5: Atypical neurofibromatous neoplasm with unknown biological potential. Figure S6: Metastasis of breast cancer. Figure S7: Lymphoma. Figure S8: Desmoid tumor. Figure S9: Myopericytoma. Figure S10: Solitary fibrous tumor. Figure S11: Amyloidoma. Figure S12: Hemangioma. Figure S13: Angiomatosis. Figure S14: Capillary hemangioma. Figure S15: Epithelioid hemangioendothelioma. Figure S16: Lymphangioma.
