# Supplementary material for: Collection of Rare Peripheral Nerve Tumors—Insights from the German Registry
Source: Cancers (Basel). 2024 Jul 20;16(14):2599. doi: 10.3390/cancers16142599 (PMC11275079; doi:10.3390/cancers16142599)

Supplementary material for:

Collection of Rare Peripheral Nerve Tumors—Insights from the German Registry

Grübel N[1], Antoniadis G[1], Uerschels AK[2], Marschal V[1], Deininger S[1], König R[1], Pala A[1], Bremer J[4], Dengler NF[3,6], Reuter M[5] , Wirtz CR[1] , Pedro MT[1], on behalf of the peripheral nerve tumor study group

[1] Peripheral Nerve Unit, Department of Neurosurgery, BKH Günzburg at Ulm University, Lindenallee 2, 89312 Günzburg, Germany

[2] Department of Neurosurgery, University Medicine Essen, Hufelandstraße 55, 45147 Essen, Germany

[3]Department of Neurosurgery, Charité University of Berlin, Charitéplatz 1, 10117 Berlin, Germany

[4] Department of Neuropathology, University of Aachen, Pauwelstraße 30, 52074 Aachen

[5] Department of Neuroradiology, BKH Günzburg at Ulm University, Lindenallee 2, 89312 Günzburg, Germany

[6] Department of Neurosurgery, Helios Hospital Bad Saarow, Pieskower Str. 33, 15526 Bad Saarow

Intrinsic PNT

Figure S1: *Hybrid nerve sheath tumor (Schwannoma/Neurofibroma).*

(a) shows a coronal T1-weighted contrast-enhanced MRI of a hybrid nerve sheath tumor of the left inferior brachial plexus (*, Schwannoma/Neurofibroma) measuring 39x41x74 mm. It reveals a heterogeneous contrast enhancement. Microsurgical gross total resection was performed. No neurological deficits pre- and postoperatively were observed. (b) Intraoperative image of the same tumor (circle). The lateral and medial fascicles of the brachial plexus are marked with yellow vessel loops. (c) Ultrasound of the same patient revealed a hypoechogenic tumorous lesion preoperatively, categorized as type C following subtype classification (Table 3).

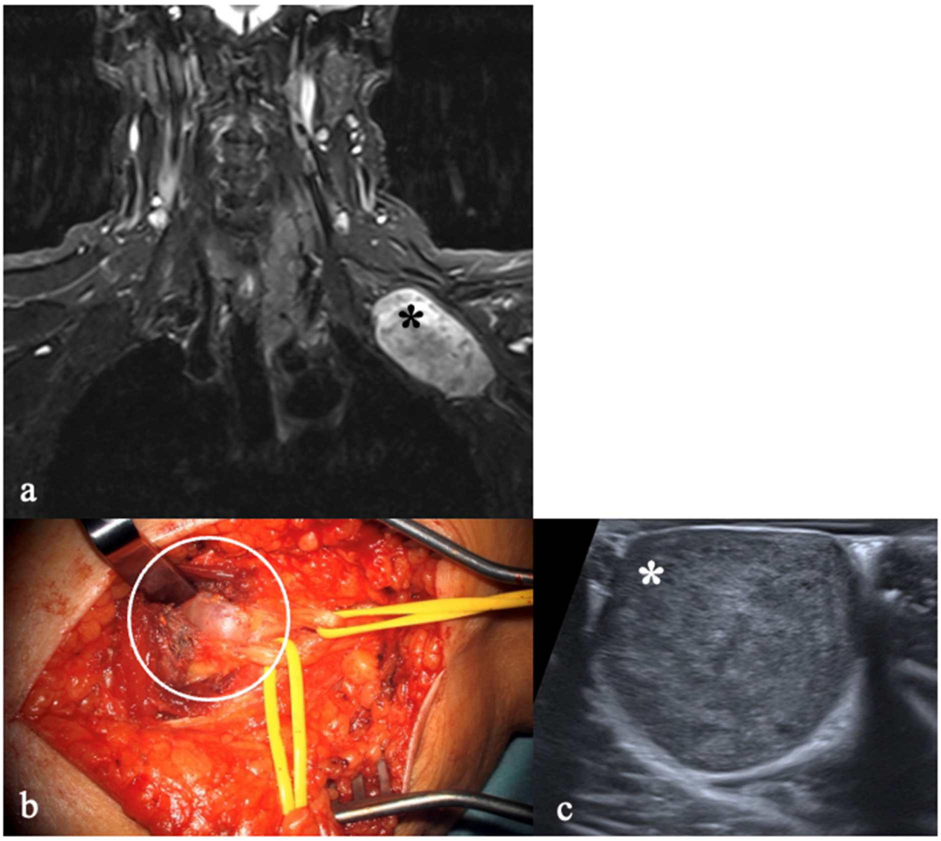

Figure S2: *Hybrid nerve sheath tumor (Schwannoma/Perineurioma).*

(a-c) shows a T1-weighted MRI, with and without contrast enhancement, of a hybrid nerve sheath tumor (Schwannoma/Perineurioma) of the cutaneous antebrachial medial nerve (arrow) measuring 29x36x71 mm. The contrast enhancement appears heterogeneous and partly cystic in T1 without contrast enhancement, while it is homogeneous in T1 without contrast enhancement. Functional fascicle-sparing microsurgical gross total resection was performed. The patient experienced preoperative sensory deficits, which did not resolve after surgery. No recurrence was observed three months postoperatively.

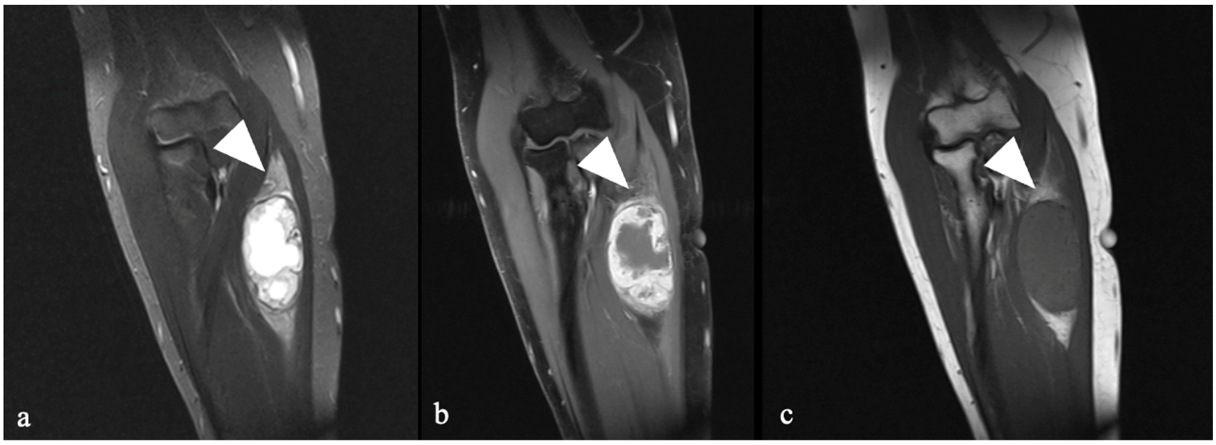

Figure S3: *Malignant peripheral nerve sheath tumor.*

Coronal (a) and axial (b) gadolinium-enhanced T1 MRI depicts a heterogeneous, cystic tumorous lesion (30x28x23 mm) of the right peroneal nerve, exhibiting partial hemorrhage and infiltration into adjacent tissue (arrow). Intraoperative imaging (c, d). Microsurgical enucleation under nerve stimulation was performed. Despite imaging suggesting infiltrative growth, microsurgical enucleation proved feasible in preserving fascicles en passant.
Subsequent histopathological analysis confirmed a malignant peripheral nerve sheath tumor (MPNST). At initial diagnosis, the patient's age was 33 years, and no neurofibromatosis disease was known.

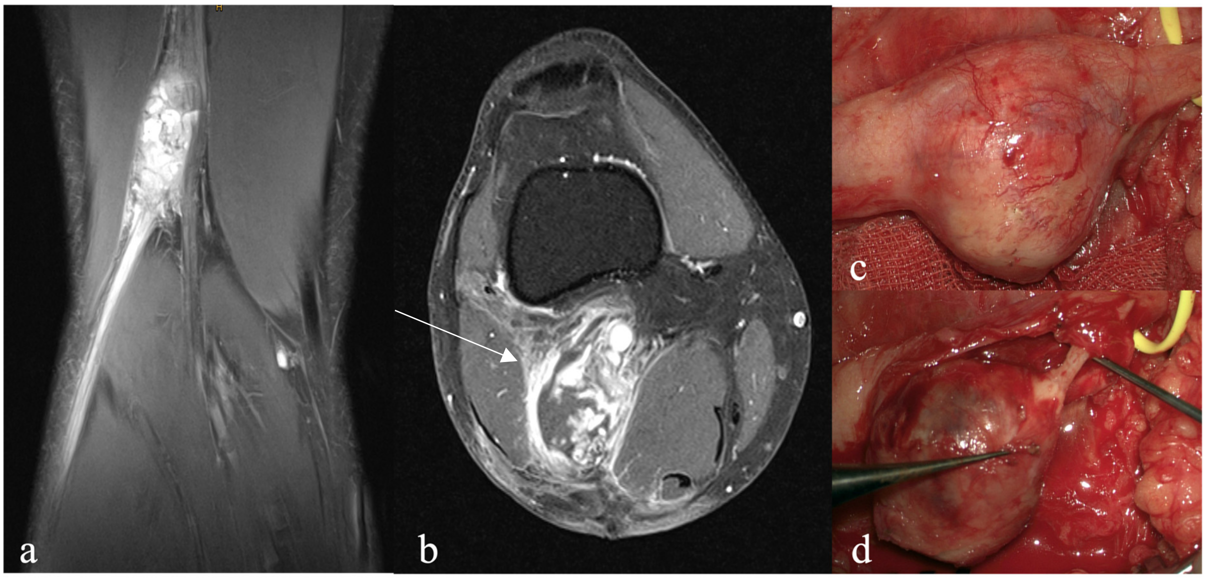

Figure S4: *Perineurioma.*

Coronal (a) and axial (b) gadolinium-enhanced T1 MRI illustrates a characteristic feature of perineurioma, presenting as a heterogeneous contrast-enhancing long-distance enlargement of a peripheral nerve (arrows). An intraoperative ultrasound revealed enlarged tumorous fascicles (arrow) adjacent to fascicles of normal size, categorized as type A following subtype classification (Table 3) (c). The surgical intervention involved a biopsy of a tumorous 'silent' motor fascicle followed by epineuriotomy under nerve stimulation (d, circle). The histopathological assessment confirmed the diagnosis of perineurioma.

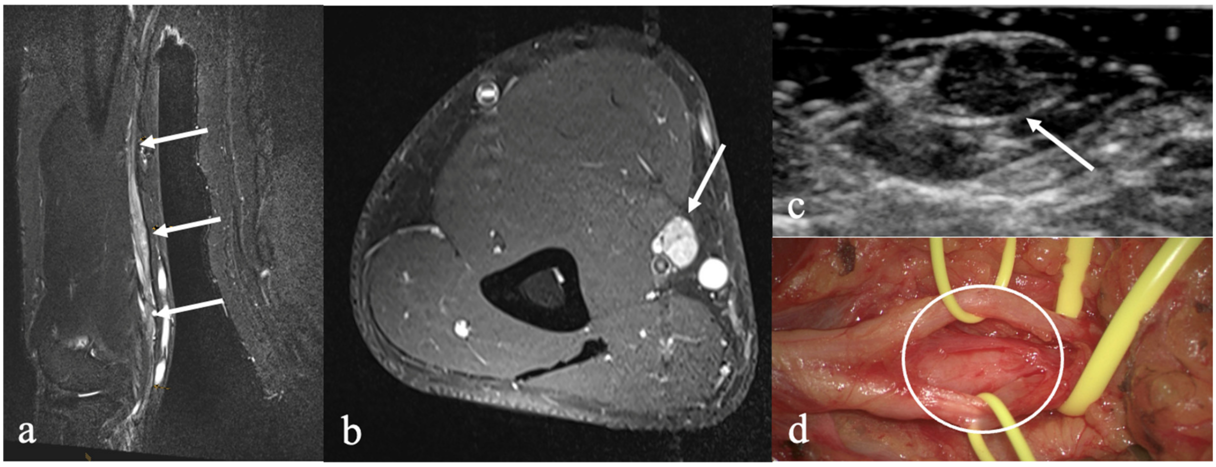

Figure S5: *Atypical neurofibromatous neoplasm with unknown biological potential.*

Image-based representation of an atypical neurofibromatous neoplasm with unknown biological potential (ANNUBP) in a 32-year-old female patient with neurofibromatosis type 1. Coronal (a) and axial (b) T1-weighted gadolinium-enhanced MRI images reveal a homogenous gadolinium-enhanced mass (*) measuring 52x24x28 mm, originating from the dorsal aspect of the sciatic nerve in the distal third of the thigh. Additionally, multiple small neurofibromas are evident throughout the sciatic nerve, intramuscularly and subcutaneously (arrow). T2-weighted MRI (d) indicates that the mass is non-cystic and homogenous. 18F-FDG-PET/CT (c) showcases FDG accumulation in the right sciatic nerve, with an SUVmean/max of 6.8/11.7.

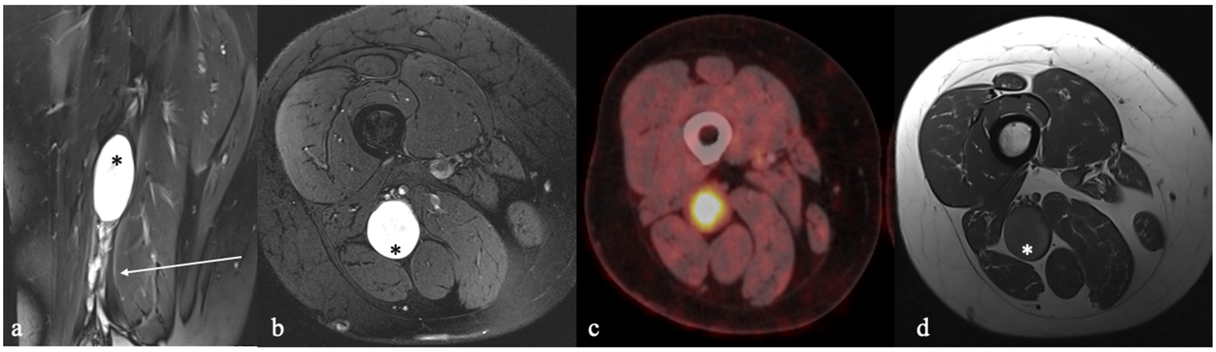

Extrinsic PNT

Figure S6: *Metastasis of breast cancer.*

Gadolinium-enhanced coronal MRI (a) reveals a 65x58x80 mm large contrast-enhanced heterogeneous mass infiltrating surrounding tissues within the left brachial plexus. A partial resection was undertaken to safeguard motor and sensory functions. Preoperative symptoms predominantly included rest and stress pain, which exhibited significant improvement postoperatively, leading to enhanced overall quality of life. Pathological examination confirmed metastasis from a previously diagnosed mamma carcinoma.

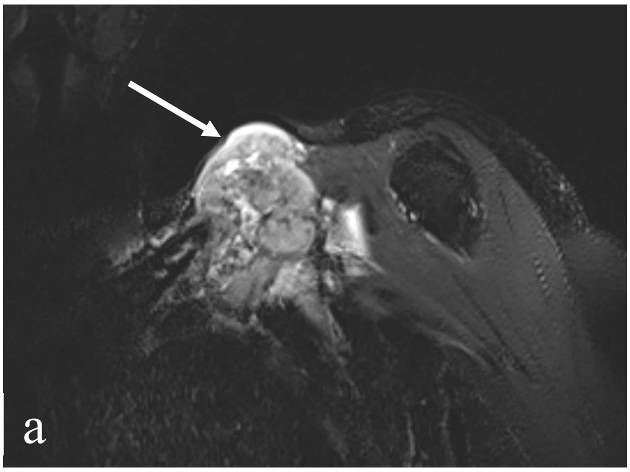

Figure S7: *Lymphoma.*

Sagittal (a) and axial (b) gadolinium-enhanced MRI of the left popliteal fossa depict a heterogeneous contrast-enhancing lesion (*) measuring 18x19x120 mm along the tibial nerve. Ultrasound image (c) demonstrates the hypoechogenicity of the lesion (*) enclosed within a circle, resembling schwannoma in appearance. After sonotypification, the tumor was classified as type C. Intraoperative images (d, e) of the same patient are provided. Microsurgical decompression of the tibial nerve, epineuriotomy, and fascicle biopsy were performed. Histopathological analysis revealed a non-Hodgkin lymphoma, with the primary disease known for one year prior. After surgery, the patient underwent radiation therapy to the left popliteal fossa and chemotherapy following the CHOP regimen as prescribed by the local tumor board.

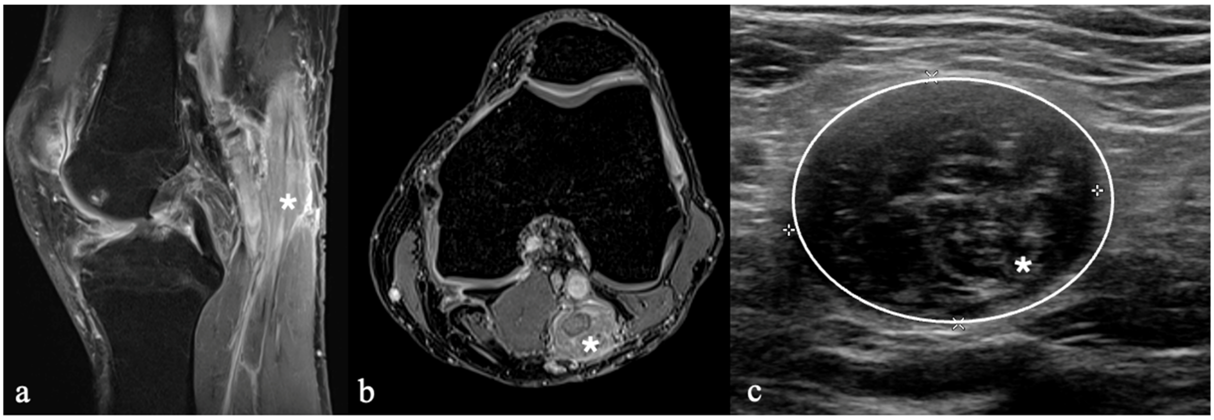

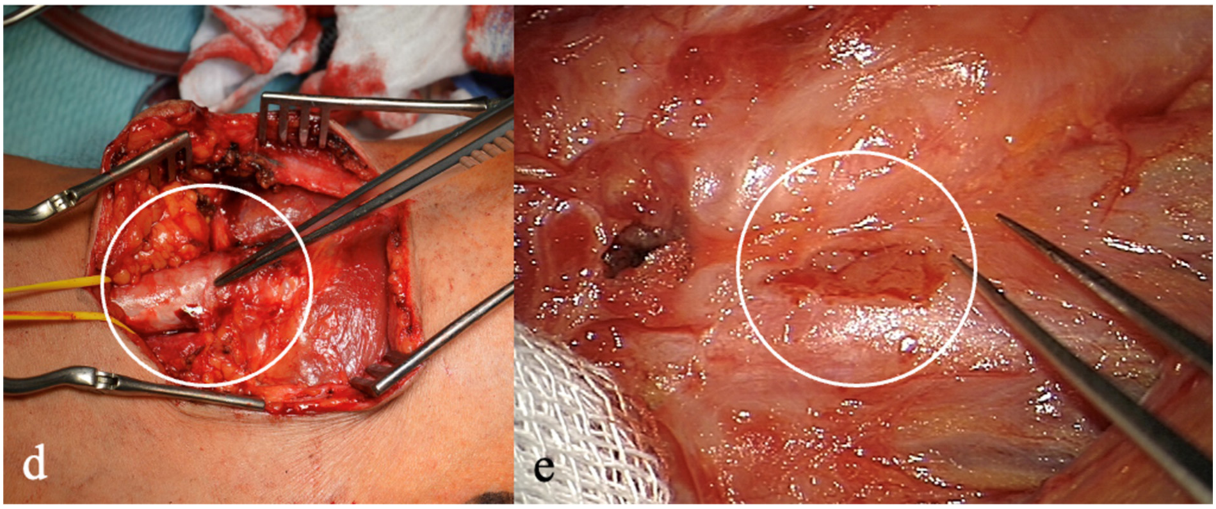

Figure S8: *Desmoid tumor.*

T2-weighted coronal (a) and axial (b) MRI images depict a diffuse tumorous mass involving the left brachial plexus, infiltrating adjacent tissues, including vessels. Intraoperative findings are illustrated in (c) (circle, C5 root is marked with a yellow vessel loop). Due to the extensive infiltration of neighboring tissue and the dense nature of the tumor, only a microsurgical partial resection was feasible surgically. The neural structures appeared to be enveloped by the tumor in a frosting-like manner. Pre- and postoperative motor and sensory deficits persisted after surgery but did not worsen. Histopathological examination confirmed the presence of a desmoid tumor involving the left brachial plexus.

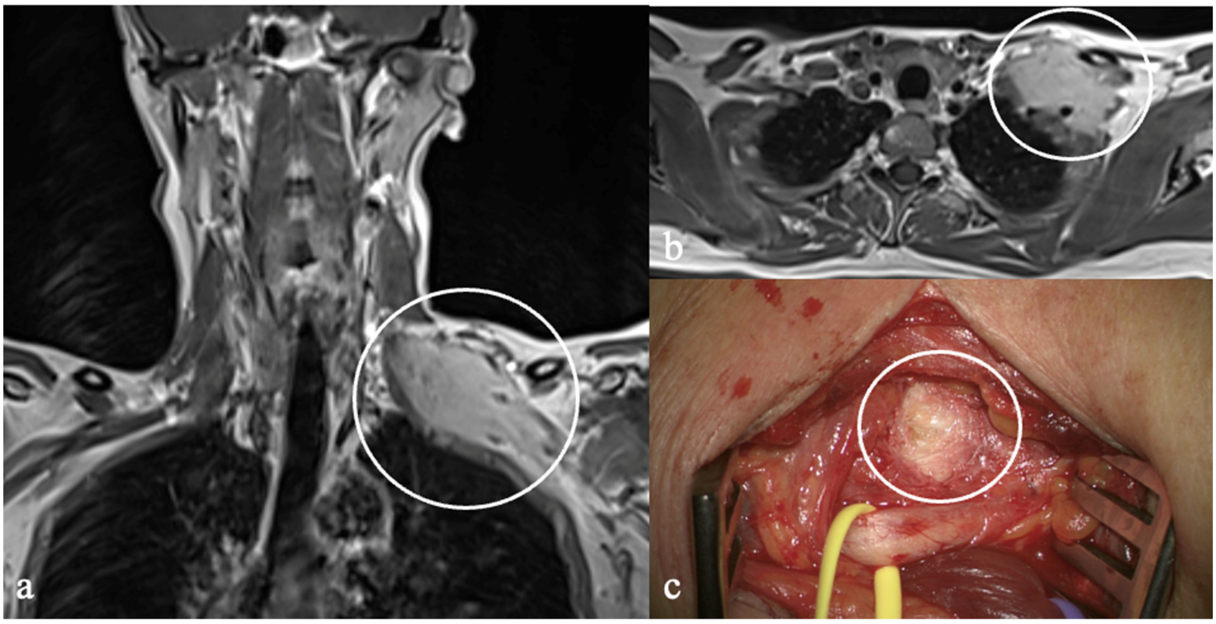

Figure S9: *Myopericytoma.*

Contrast-enhanced T1-weighted MRI images in coronal (a) and axial (b) views of the right arm reveal a small, homogeneously enhanced lesion (arrow) measuring 10x8x5mm attached to the cutaneous antebrachial lateral nerve. Intraoperative imaging of the lesion is depicted in (c, d). Complete microsurgical resection was conducted, and histopathological analysis confirmed the diagnosis of Myopericytoma. No recurrence was observed after 3 months.

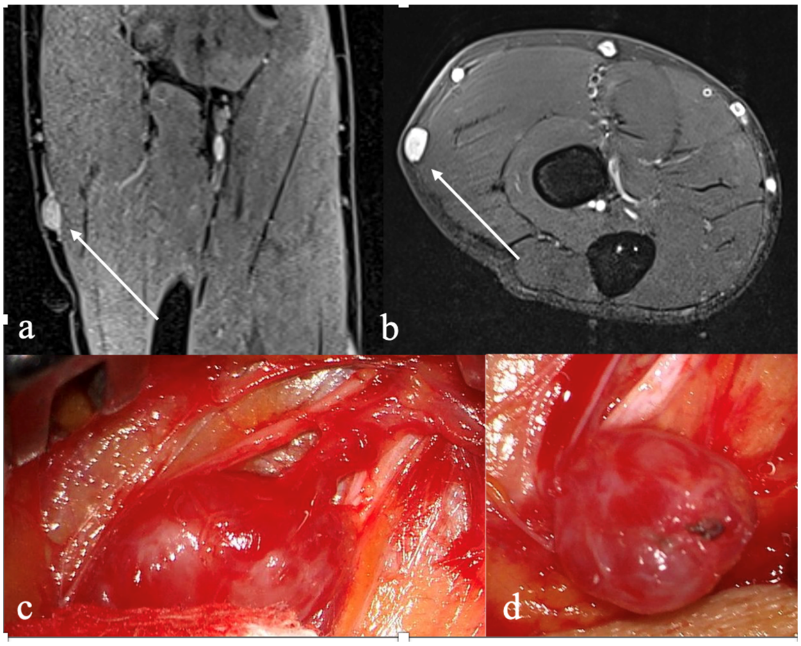

Figure S10: *Solitary fibrous tumor.*

Contrast-enhanced T1-weighted MRI images depict two patients with histologically confirmed solitary fibrous tumors (SFT) in the lower extremity. Both tumors exhibit spindle-shaped morphology and demonstrate strong, homogeneous contrast enhancement. Coronal (a), axial (b), and intraoperative (c,*) images illustrate an SFT associated with the right lumbosacral plexus, measuring 57x43x31 mm. Axial (d) and sagittal (e) MRI images display an SFT associated with the left sciatic nerve, measuring 29x17x21 mm. Microsurgical gross total resection was performed in both cases. Initial symptoms prompting diagnosis included rest and stress resolving post-surgery in both instances. Follow-up examinations at 14 and 3 months revealed no recurrence.

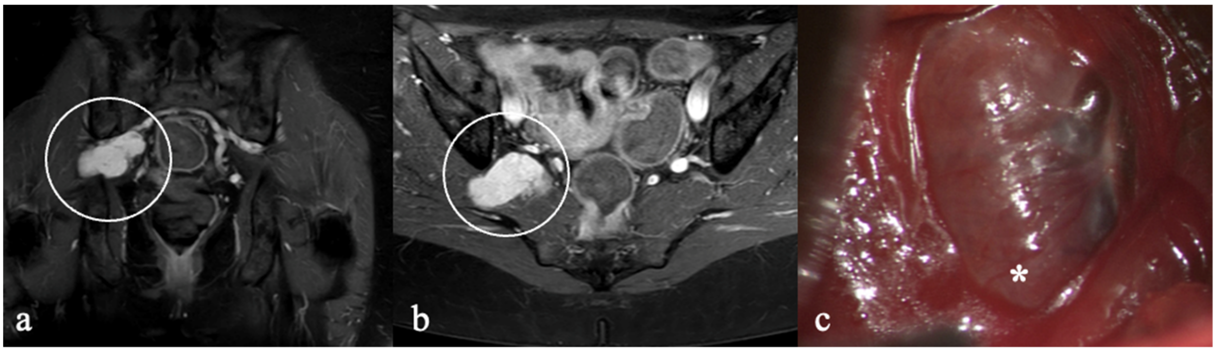

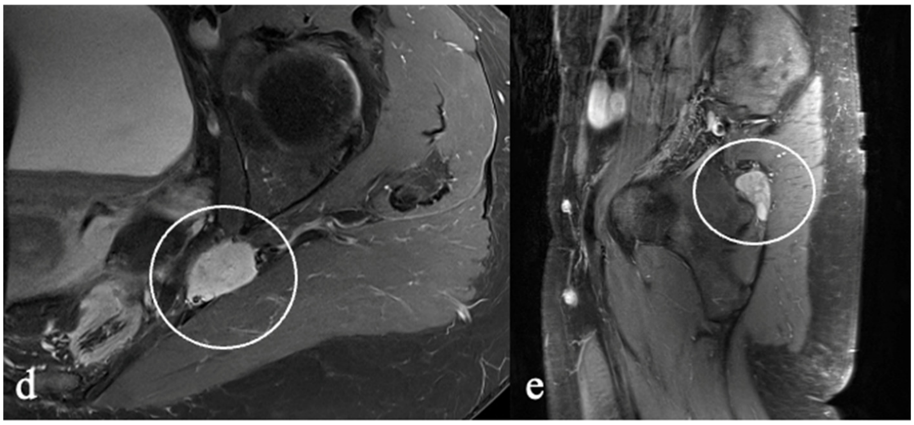

Figure S11: *Amyloidoma.*

(a) Sagittal T1 gadolinium-enhanced MRI displays a uniformly contrast-enhanced mass (*) surrounding the ulnar nerve. T2 MRI reveals a partially heterogeneous mass (b, indicated by arrow). (c) Ultrasound exhibits an intraneural hypoechogenic lesion. Intraoperative image (d) displays enlarged fascicles resembling vitreous material (*) adjacent to the functional nerve fascicle (triangle). A biopsy was conducted under nerve stimulation. Histopathological analysis confirmed the presence of an amyloidoma. Reference pathology in Kiel, Germany, corroborated these findings, revealing myxoid loosened tissue (right ulnar nerve) with vascular and interstitial AL amyloid deposits of lambda light chains.

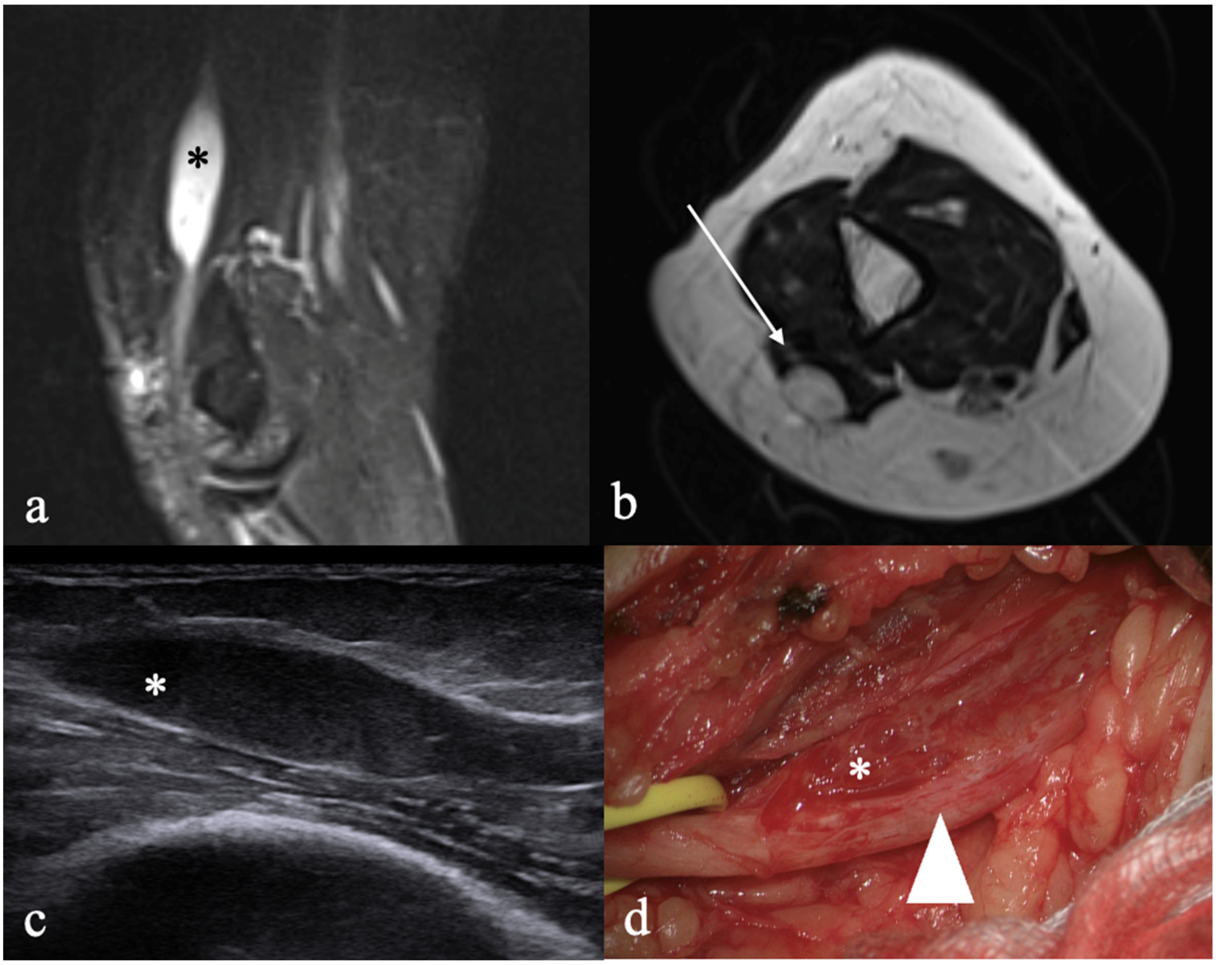

Figure S12: *Hemangioma.*

(a) Coronal and (b) axial contrast-enhanced T1 MRI of the left upper arm shows a widespread heterogeneous contrast-enhanced lesion (arrow, circle) of the median nerve in a 51-year-old female patient. The lesion also shows heterogeneous aspects in (c) axial T2 MRI. (d) Visualization of the territory of the left brachial artery using digital subtraction angiography technique. Here, a circumscribed low-flow hemangioma with a single feeder (arrow) from the brachial artery is demonstrated. Initially, an open biopsy of the mass was performed externally, which did not reveal any evidence of a tumor. Subsequently, there were progressive pain symptoms and increasing sensory and motor deficits.
The following surgical procedure was performed as a microsurgical resection of the approximately 10 cm-long intraneural hemangioma from the median nerve (e). Intraoperatively, an intraneural, interfascicular vascular-rich space-occupying lesion was observed, which could not be resected while preserving the nerve (e-g). According to the preoperative agreement with the patient, nerve transplantation was performed by autologous transplantation with 5x14 cm long sural nerve interpositions (h).

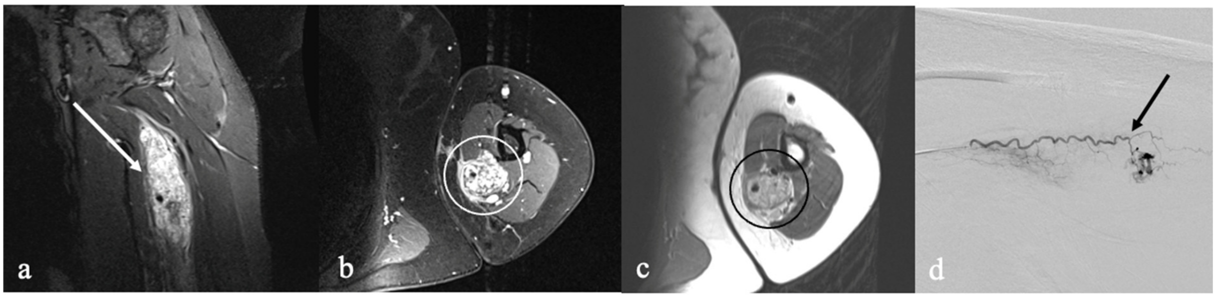

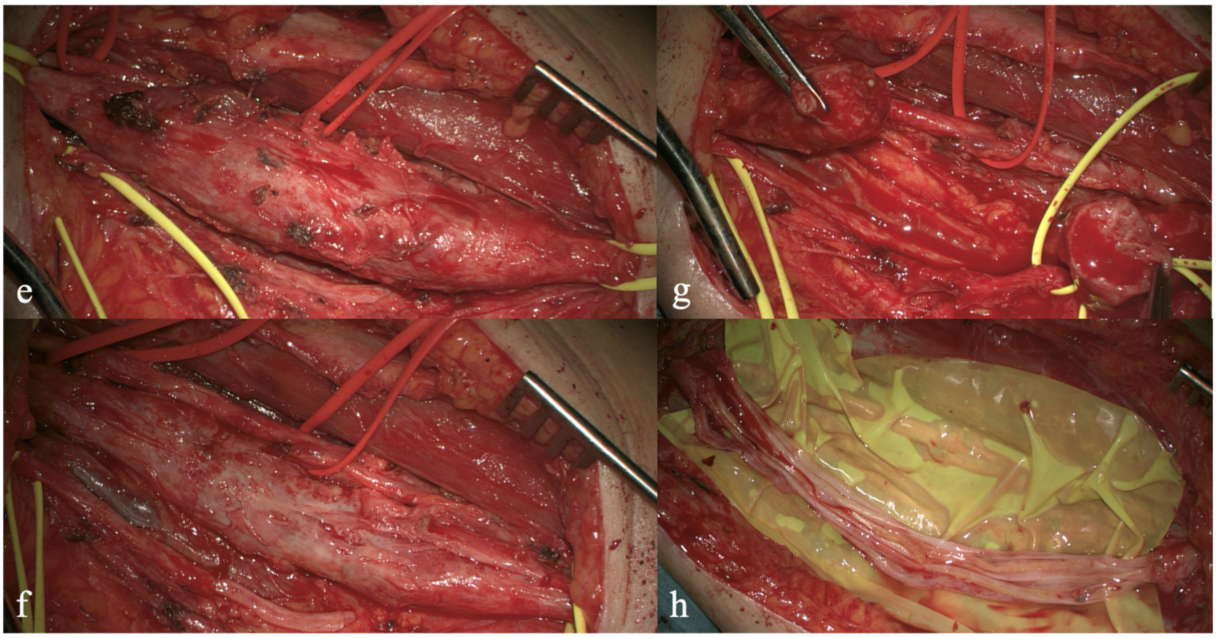

Figure 13: *Angiomatosis.*

(a) Contrast-enhanced coronal MRI shows a small (7x4x12 mm) lesion (circle and arrow) in contact with the peroneal nerve without contrast enhancement. (b, c) the sonographic representation shows enlarged fascicles (circles), thereby type A. Intraoperative imaging (d) shows a tumorous lesion (arrow), as suspected on ultrasound. Both sides of the peroneal nerve

are marked with vessel loops. After microsurgical interfascicular neurolysis, (e) displays the fan-shaped arrangement of the space-occupying lesion concerning each fascicle. Partial microsurgical resection of the extraneural parts, interfascicular neurolysis, and a fascicle biopsy were performed. The histopathological diagnosis was angiomatosis of the peroneal nerve.

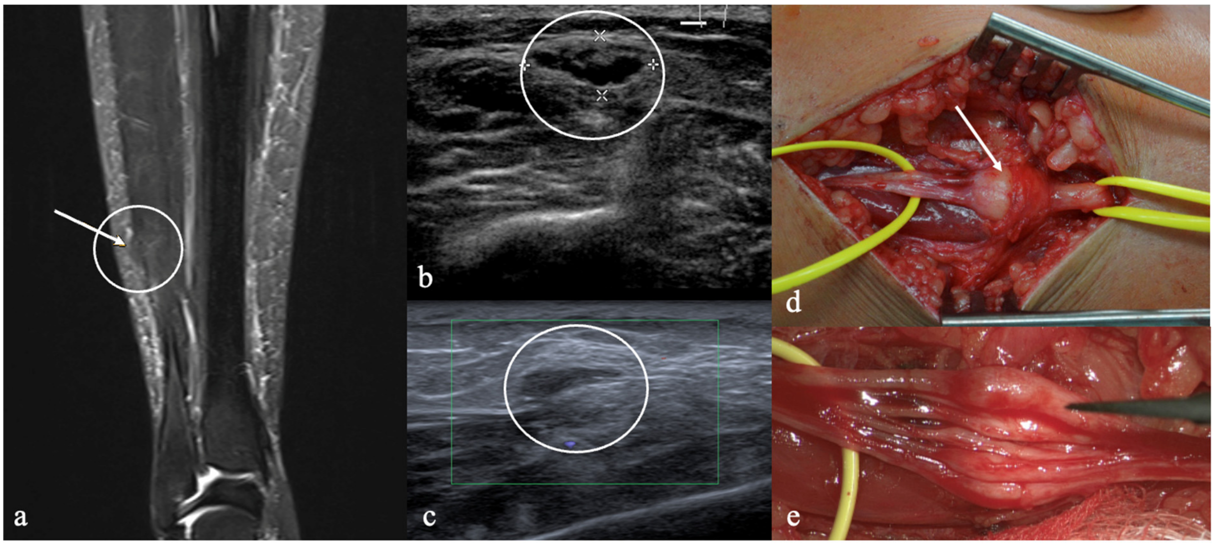

Figure S14: *Capillary hemangioma.*

(a) Axial T1 contrast-enhanced MRI shows a small (6x3x7 mm) non-contrast enhanced lesion (circle and arrow) of the left sciatic nerve. (b) Intraoperative imaging. An extensively epineuriotomy of the sciatic nerve was performed. The peroneal and tibial parts of the sciatic nerve are marked with a yellow vessel loop in the intraoperative image. The biopsy site was taken of a lateral fascicle, silent after nerve stimulation and is marked with a circle. Histopathological examination revealed an intraneural capillary hemangioma. The patient was a 21-year-old male with motor and sensory deficits leading to diagnosis. Six months after diagnosis, there is no evidence of disease progression.

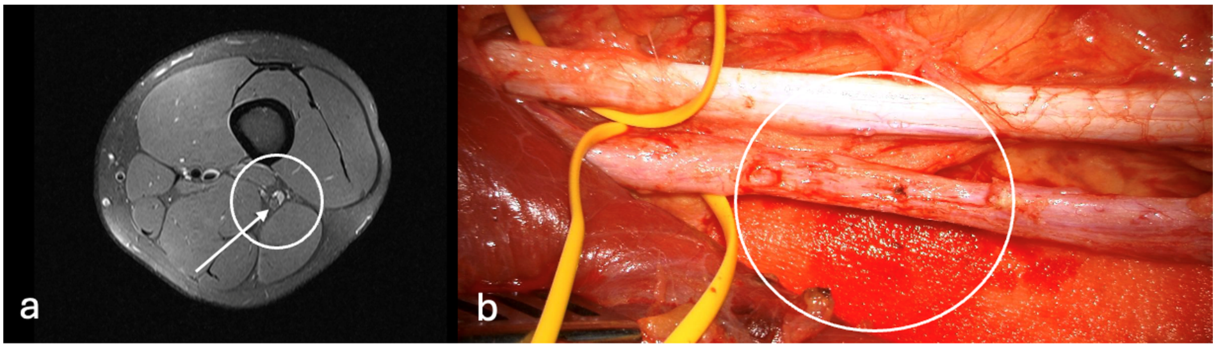

Figure S15: *Epithelioid hemangioendothelioma.*

(a) A contrast-enhanced coronal MRI revealed an inhomogeneous lesion along the vascular nerve sheath (circle). The histopathological evaluation confirmed an epithelioid hemangioendothelioma associated with the cutaneous antebrachial medial nerve. ^{18}F-FDG-PET/CT scans (b, c) depicted FDG accumulation along the cutaneous antebrachial medial nerve (arrow), with a SUVmean/max of 4/7.1. Microsurgical gross total resection was conducted under nerve stimulation, preserving functional fascicles. No neurological deficits were

observed before or after surgery. Six months post-resection, there was no evidence of recurrence, and no adjuvant treatment was administered.

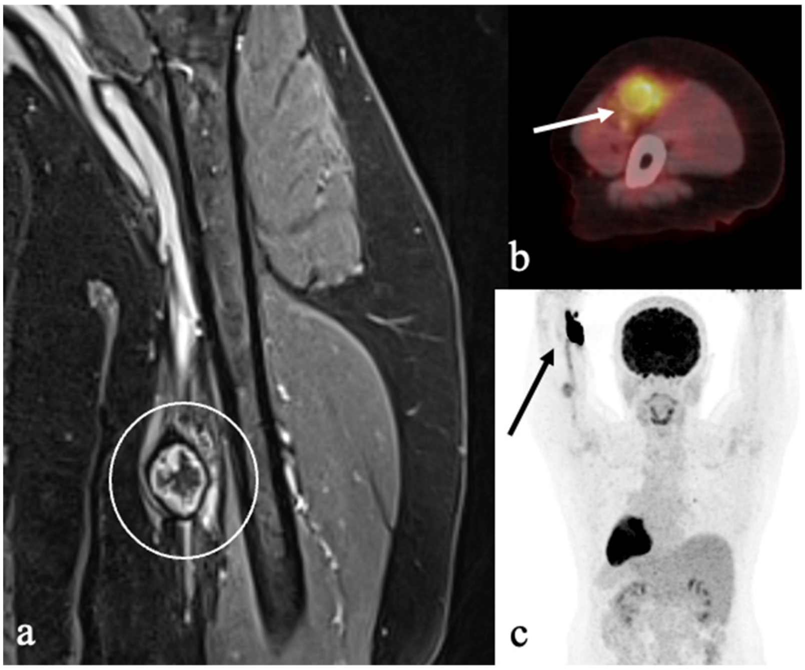

Figure S16: *Lymphangioma*

T1 (a) and T2 (b) MR images of the left elbow of a 24-year-old woman reveal a diffuse, honeycomb-like tumorous mass within the median nerve (arrows, circle). Intraoperative imaging confirmed the extensive intraneural involvement. The surgical intervention consisted of a biopsy (*) and decompression of the median nerve (c, d). Histopathological examination confirmed the diagnosis of an intraneural lymphangioma. Initial symptoms prompting diagnosis included stress-related pain and persistent motor and sensory deficits. The patient remains clinically and radiologically stable. Follow-up examinations are conducted using ultrasound.

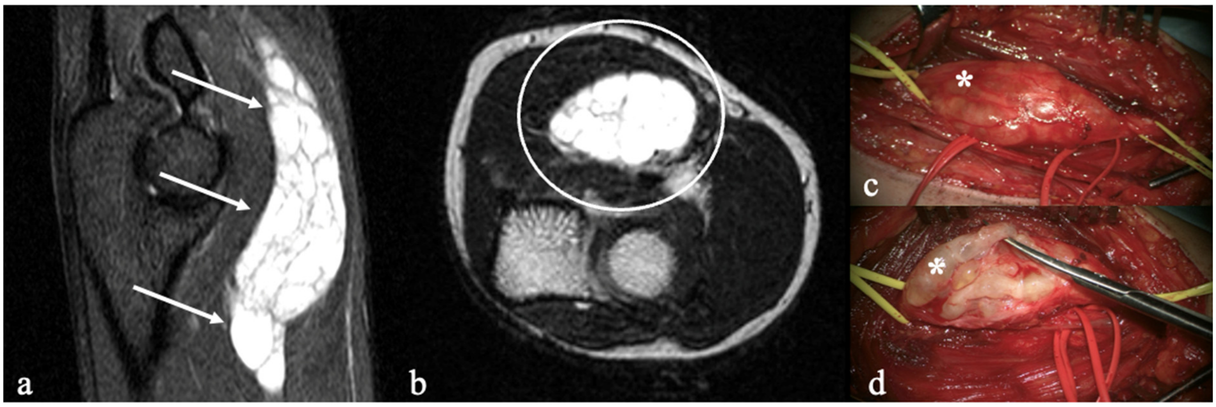

Supplement: Supplementary file 1 [file cancers-16-02599-s001.zip › cancers-3093230-supplementary.pdf]
